# Mental health service use and associated predisposing, enabling and need factors in community living adults and older adults across Canada

**DOI:** 10.1186/s12913-023-09335-5

**Published:** 2023-04-12

**Authors:** Helen-Maria Vasiliadis, Jessica Spagnolo, Marie-Josée Fleury, Jean-Philippe Gouin, Pasquale Roberge, Mary Bartram, Sébastien Grenier, Grace Shen-Tu, Jennifer E. Vena, JianLi Wang

**Affiliations:** 1 Département des sciences de la santé communautaire, Université de Sherbrooke, 2500, boul. de l’Université, J1K 2R1 Sherbrooke (Québec), Canada; 2 Centre de recherche Charles-Le Moyne, 150, place Charles‑Le Moyne, C. P. 200, J4K 0A8 Longueuil (Québec), Canada; 3 Douglas Mental Health University Institute, 6875 boul., H4H 1R3 LaSalleVerdun (Québec), Canada; 4 McGill University, 845 Sherbrooke St W, H3A 0G4 Montreal (Québec), Canada; 5 Department of Psychology, Concordia University, 7141 Sherbrooke West, H4B 1R6 Montreal (Québec), Canada; 6 Centre de recherche de l’Institut universitaire de gériatrie de Montréal, 4545 Queen Mary Rd, H3W 1W6 Montreal (Québec), Canada; 7 Département de médecine familiale et d’urgence, Université de Sherbrooke, 2500, boul. de l’Université, J1K 2R1 Sherbrooke (Québec), Canada; 8 Centre de recherche du Centre hospitalier universitaire de Sherbrooke, 12e Avenue N Porte 6, J1H 5N4 Sherbrooke (Québec), Canada; 9 Mental Health Commission of Canada, 350 Albert St #1210, K1R 1A4 Ottawa (Ontario), Canada; 10 School of Public Policy & Administration, Carleton University, 1125 Colonel By Drive, K1S 5B6 Ottawa (Ontario), Canada; 11 Département de psychologie, Université de Montréal, 90, avenue Vincent d’Indy, H2V 2S9 Montréal (Québec), Canada; 12 Alberta’s Tomorrow Project, Cancer Research & Analytics, Cancer Care Alberta, Alberta Health Services, 1820 Richmond Road SW, T2T 5C7 Calgary (Alberta), Canada; 13 Department of Community Health and Epidemiology, Dalhousie University, 5790 University Avenue, B3H 1V7 Halifax (Nova Scotia), Canada

**Keywords:** Depression, Anxiety, Mental health service use, Health factors, Inequities, COVID-19, Canada

## Abstract

**Objectives:**

Using Andersen’s model of health care seeking behavior, we examined the predisposing, enabling, and need factors associated with mental health service use (MHSU) during the first wave of the COVID-19 pandemic across Canada.

**Methods:**

The sample included n = 45,542 participants in the 5 established regional cohorts of the Canadian Partnership for Tomorrow’s Health (CanPath) and who responded to the CanPath COVID-19 health survey (May-December 2020), with complete data on MHSU. Multivariable logistic regression analyses were carried out to study MHSU as a function of predisposing, enabling, and need factors. Analyses were stratified by regional cohort.

**Results:**

Among the need factors, individuals reporting moderate/severe symptoms of depression and anxiety and poorer self-rated mental health were more likely to report MHSU. Among the enabling factors, receipt of informational/financial/practical support was associated with increased MHSU. While income was not consistently associated with MHSU, reported decrease in income was marginally associated with reduced MHSU. Among the predisposing factors, identifying as female or other gender minority was associated with increased MHSU, as was the presence of past-year cannabis use. In contrast, older age and alcohol consumption were associated with reduced MHSU.

**Conclusion:**

Need factors were consistently associated with MHSU. Although income inequities in MHSU were not observed, changes such as reduced income during the pandemic may lead to barriers in accessing mental health services. Future research should focus on better identifying contextual enabling factors and policies that overcome financial barriers to MHSU.

## Introduction

Canadian population-based studies prior to the pandemic showed past-year prevalence estimates of mental health service use (MHSU) between 9.5% and 19.4% [1, 2]. During the pandemic, mental health service disruptions were reported and face-to-face medical consultations [3–5] were replaced with telehealth consultations [5–9]. Data from the 2021 Survey on COVID-19 and Mental Health (SCMH) showed that 10% of Canadian respondents were currently communicating with a professional for their mental health [10]. In the USA, reports from the 2020 cross-sectional Household Pulse Survey (HPS) showed past-month prevalence estimates for mental health counselling between 9 and 10% [11] and that close to 13% of participants reported a past-month unmet mental health need for counselling [12].

According to Andersen’s model [13, 14] of health service use can be explained by predisposing (e.g. socio-demographic factors, health beliefs), enabling (e.g. economic, psychosocial, resources) and need (e.g. health status) factors. Equitable access has been defined as when health service use is explained by need and demographic factors [13]. Inequity would exist if MHSU was associated with factors other than need [15]. In a systematic review using Andersen’s socio-behavioural model, need was the most consistent in explaining MHSU and included perceived health status, disability and comorbidity [16]. Enabling factors not associated with MHSU were income and rural residence; while findings for unemployment were inconsistent [16]. The predisposing factors associated with MHSU, although not observed across all studies, included female gender, being Caucasian, reporting a higher education and not being married [16].

During the pandemic, the factors associated with increased MHSU in the USA HPS were race/ethnicity (White, in comparison to Black, Hispanic and Asian), female sex, single adults with and without children, and higher education [11]. In Canada, data from the SCMH in early 2021 showed a higher proportion of women than men, adults than seniors, and Caucasians than non-Caucasians, that reported currently communicating with a professional for their mental health [10]. Other studies also highlighted that the pandemic reinforced pre-existing social and health inequities [17–19]. Scarce are the studies that have reported on the need factors associated with MHSU in general populations studies during the pandemic.

Informing on the presence of equitable access to mental health services during the pandemic is important [20–23] as this can contribute to inform health policies related to resource allocation and ultimately respond to population mental health needs irrespective of socio-economic status. Using Andersen’s model [14] of healthcare seeking behavior, we aimed to assess the predisposing (e.g., age, gender, lifestyle behaviours), enabling (e.g., income), and need (e.g., perceived health) factors associated with MHSU during the first wave of the pandemic. We hypothesize that need and predisposing factors would be associated with MHSU, while enabling factors such as income would not be associated with MHSU.

## Methods

### Survey data

This study relied on harmonized data from 45,542 adults with complete data who had participated in the previous 2018 follow-up and 2020 COVID health survey, early in the COVID-19 pandemic (May to December 2020), in the 5 established regional cohorts of the Canadian Partnership for Tomorrow’s Health (CanPath) (formerly Canadian Partnership for Tomorrow Project, CPTP) [24]. Details on CanPath regional cohorts have been published elsewhere [25]. In brief, cohort participants were from Atlantic Partnership for Tomorrow’s Health (Atlantic PATH), Quebec’s CARTaGENE (CaG), Ontario Health Study (OHS), Alberta’s Tomorrow Project (ATP), and British Columbia Generations Project (BCGP) regional cohorts. As compared to the general Canadian population, the cohort participants were more likely women, more likely to report having attained a graduate degree and less likely to report less than a high school education, more likely to be retired and less likely to be unable to work or unemployed. The proportion of self-reported common chronic diseases (e.g. hypertension, arthritis, asthma and diabetes) were similar to those found in Canadian general population samples [25]. The study was approved by the institutional research ethics board of the CISSS Montérégie-Centre (#2021 − 563).

## Measures

### Dependent variable

MHSU was assessed in the CanPath COVID 2020 health survey, between May and December 2020, by asking respondents at time of interview whether they had accessed mental health services since March 2020. Participants responding either ‘Yes – using resources already in place,’ or ‘Yes - initiated new use of services’ were combined and categorized as yes, and those responding ‘No’ were categorized as no. Respondents answering yes, were also asked if mental health service use was for any of the following conditions: anxiety, depression, stress, other.

### Independent variables

#### Predisposing factors

Predisposing factors assessed in the 2020 survey included age, categorized into groups (35–44 years, 45–54 years, 55–64 years, ≥ 65 years); gender identity (male, female, cultural gender minority (e.g., two-spirit), other (e.g., non-binary, gender fluid, no answer)). Information on race was ascertained in the 2018 follow-up survey. Race/ethnicity was categorized as whether the respondent identified as a visible minority, ‘no’ if Caucasian (White), and ‘yes’ if non-Caucasian (Arab, Black, East Asian, Filipino, Jewish, Latin American/Hispanic, South Asian, South-East Asian, West-Asian, Other). Last, lifestyle habits included smoking in the past month (not at all, occasionally/daily); past-year cannabis use (yes/no); past-year average weekly alcohol consumption (never, less than daily (≤ 5 times/week), almost daily (6–7 times/week)).

#### Enabling factors

Enabling factors included the regional cohort in which each participant was initially recruited; total household income before taxes (previous year); self-reported decrease in income during the pandemic (yes/no); whether respondents worked as a medical professional (e.g. physician, nurse, hospital employee, first responder, pharmacist, with exposure to patients, yes/no) or an essential service provider (e.g. grocery store attendant, public transit, police, security, etc.) with regular exposure to members of the public, yes/no); and receipt of informational/financial/practical support (housing/childcare/food delivery/material goods furniture/clothing, yes/no). Respondents were asked about delays in seeing a healthcare professional for a new problem (categorized as yes/no, not applicable/not answered).

#### Need factors

Measures on the presence of moderate to severe symptoms of anxiety and depression were available in both the 2020 COVID-19 survey and from an earlier pre-pandemic follow-up survey carried out in 2018 [24, 25]. Anxiety was assessed in both surveys with the seven-item Generalized Anxiety Scale (score range 0–21) and a positive screen for anxiety was based on the presence of moderate to severe symptoms of anxiety according to a cut-off ≥ 10 [26]. A positive screen of depression was based on the presence of moderate to severe symptoms of depression according to a cut-off ≥ 10 on the eight-item Patient Health Questionnaire Depression Scale (PHQ-8, score range 0–24) [27]. A dummy variable with 4 levels was created to summarize the positive screen for anxiety and depression at each survey and categorized as follows: no positive screen for anxiety and depression at either survey; remitted (positive screen in 2018, but not 2020); incident (positive screen in 2020, but not in 2018); and persistent (positive screen in 2018 and 2020).

The following need factors were assessed in the 2020 COVID-19 survey. Self-rated current emotional/mental health (poor/fair/good versus very good/excellent) and change in emotional/mental health since the pandemic (worse, no change, better) were also assessed. Physical multimorbidity was based on self-reported lifetime physician diagnosis of cancer, diabetes, heart and circulatory conditions, cardiovascular disorder, respiratory system conditions, gastrointestinal diseases, liver or pancreatic conditions, renal disease, kidney conditions, neurological conditions, bone and joint conditions, and immune system conditions. Presence of multimorbidity was categorised as: no chronic physical or neurologic conditions; 1–3 chronic conditions; and ≥ 4 chronic conditions. Self-reported increase in alcohol consumption (yes/no) since March 2020 was also assessed.

### Data analyses

Descriptive statistics and group comparisons were based on Chi-square statistics. Bivariate and multivariable logistic regression analyses were carried out to study MHSU as a function of predisposing, enabling, and need factors. Analyses were also stratified by regional cohort. Odds Ratios (OR) and 95% confidence intervals (CI) were computed to determine the strength of associations. All adjusted OR (aOR, 95% CI) estimates presented were adjusted for all study predisposing, enabling, and need factors. C-statistics for models are also presented. Analyses were carried out using SAS version 9.4 [28].

## Results

### Sample characteristics

Characteristics of the study sample are presented in Table 1. The overall prevalence of MHSU was 6.3%, with 4.8% of respondents reporting use of services already in place and 1.5% initiating use of new services. Among individuals reporting MHSU, 46.7% were for reasons of anxiety, 44.3% for depression, 44.9% for stress, and 14.5% for other reasons.

**Table 1:** Characteristics of study sample and bivariate analyses of the association between mental health service use and predisposing, enabling and need factors

	N (%)	Mental health service use N = 45,542		p-value	Bivariate analyses
		No N = 42 683 (93.7%)	Yes N = 2 859 (6.3%)		OR_CRUDE_ (95% CI)
***Mental health service use***					
* Yes – using resources already in place*			2181 (4.8%)		
* Yes - initiated new use of services*			678 (1.5%)		
***Predisposing factors***					
CanPath Regional cohorts				< 0.0001	
British Columbia Generations Project (BCGP)	13 786 (30.3%)	12 850 (93.2%)	936 (6.8%)		1.64 (1.41. 1.90)
Alberta’s Tomorrow Project (ATP)	7 518 (16.5%)	7 031 (93.5%)	487 (6.5%)		1.56 (1.32, 1.83)
Ontario Health Study (OHS)	9 652 (21.2%)	9 057 (93.8%)	595 (6.2%)		1.48 (1.26, 1.73)
Quebec’s CARTaGENE (CaG)	5 186 (11.4%)	4 965 (95.7%)	221 (4.3%)		1.00
Atlantic PATH	9 400 (20.6%)	8 780 (93.4%)	620 (6.6%)		1.59 (1.36, 1.86)
Age group				< 0.0001	
< 44 years	1 550 (3.4%)	1 304 (84.1%)	246 (15.9%)		1.00
45–54 years	6 881 (15.1%)	6 060 (88.1%)	821 (11.9%)		0.72 (0.62, 0.84)
55–64 years	14 908 (32.7%)	13 862 (93.0%)	1 046 (7.0%)		0.40 (0.34, 0.47)
≥ 65 years	22 203 (48.8%)	21 457 (96.6%)	746 (3.4%)		0.18 (0.16, 0.22)
Gender identity				< 0.0001	
Male	15 447 (33.9%)	14 865 (96.2%)	582 (3.8%)		1.00
Female	29 974 (65.8%)	27 712 (92.5%)	2 262 (7.6%)		2.09 (1.90, 2.29)
Other gender minority	121 (0.3%)	106 (87.6%)	15 (12.4%)		3.62 (2.09, 6.25)
Race/ethnicity (visible minority)				0.84	
Caucasian	43 190 (94.8%)	40 481 (93.7%)	2 709 (6.3%)		0.98 (0.83, 1.16)
Non-Caucasian	2 352 (5.2%)	2 202 (93.6%)	150 (6.4%)		1.00
Current smoking				< 0.0001	
Yes	1 823 (4.0%)	1 663 (91.2%)	160 (8.8%)		1.46 (1.24, 1.73)
No	43 719 (96.0%)	41 020 (93.8%)	2 699 (6.2%)		1.00
Past-year cannabis use				< 0.0001	
Yes	5 871 (12.9%)	5 226 (89.0%)	645 (11.0%)		2.09 (1.90, 2.29)
No	39 671 (87.1%)	37 457 (94.4%)	2 214 (5.6%)		1.00
Past year average weekly alcohol consumption				< 0.0001	
Never	5 140 (11.3%)	4 704 (91.5%)	436 (8.5%)		1.00
Less than Daily (≤ 5 times/week)	33 967 (74.6%)	31 834 (93.7%)	2 133 (6.3%)		0.72 (0.65, 0.81)
Almost daily (Six to seven times a week	6 435 (14.1%)	6 145 (95.5%)	290 (4.5%)		0.51 (0.44, 0.59)
***Enabling factors***					
Total household income in year prior to pandemic				< 0.0001	
< $24,999	1 457 (3.2%)	1 298 (89.1%)	159 (10.9%)		1.00
$25,000 - $49,999	5 255 (11.5%)	4 932 (93.9%)	323 (6.1%)		0.54 (0.44, 0.65)
$50,000 - $74,999	7 696 (16.9%)	7 242 (94.1%)	454 (5.9%)		0.51 (0.42, 0.62)
$75,000 - $99,999	7 318 (16.1%)	6 887 (94.1%)	431 (5.9%)		0.51 (0.42, 0.62)
$100,000 - $149,999	8 661 (19.0%)	8 071 (93.2%)	590 (6.8%)		0.60 (0.50, 0.72)
≥ $150,000	7 559 (16.6%)	7 017 (92.8%)	542 (7.2%)		0.63 (0.52, 0.76)
Prefer not to answer	7 147 (15.7%)	6 820 (95.4%)	327 (4.6%)		0.39 (0.32, 0.48)
Missing response	449 (1.0%)	416 (92.7%)	33 (7.3%)		0.65 (0.44, 0.96)
Decrease in income during pandemic				< 0.0001	
Yes	10 264 (22.5%)	9 446 (92.0%)	818 (8.0%)		1.41 (1.30, 1.53)
No	35 278 (77.5%)	33 237 (94.2%)	2 041 (5.8%)		1.00
Medical or other professional with exposure to patients				< 0.0001	
Yes	2 739 (6.0%)	2 492 (91.0%)	247 (9.0%)		1.53 (1.33, 1.75)
No	42 803 (94.0%)	40 191 (93.9%)	2 612 (6.1%)		1.00
Essential worker with exposure to public				< 0.0001	
Yes	2 818 (6.2%)	2 592 (92.0%)	226 (8.0%)		1.33 (1.15, 1.53)
No	42 724 (93.8%)	40 091 (93.8%)	2 633 (6.2%)		1.00
Informational/financial/practical support received				< 0.0001	
Yes	11 315 (24.9%)	10 250 (90.6%)	1 065 (9.4%)		1.88 (1.74, 2.03)
No	34 227 (75.2%)	32 433 (94.8%)	1 794 (5.2%)		1.00
Delay in health care for new health problem				< 0.0001	
Yes	5 108 (11.2%)	4 540 (88.9%)	568 (11.1%)		2.03 (1.80, 2.29)
No	9 976 (21.9%)	9 397 (94.2%)	579 (5.8%)		1.04 (0.94, 1.14)
Not applicable/not answered	30 458 (66.9%)	28 746 (94.4%)	1 712 (5.6%)		1.00
***Need factors***					
Pattern of moderate – severe symptoms of anxiety^§^				< 0.0001	
No moderate-severe symptoms	41 364 (90.8%)	39 415 (95.3%)	1 949 (4.7%)		1.00
Remitted	1 745 (3.8%)	1 504 (86.2%)	241 (13.8%)		3.24 (2.81, 3.74)
Incident	1 680 (3.7%)	1 263 (75.2%)	417 (24.8%)		6.68 (5.92, 7.53)
Persistent	753 (1.7%)	501 (66.5%)	252 (33.5%)		10.17 (8.69, 11.91)
Pattern of moderate – severe symptoms of depression^§^				< 0.0001	
No moderate-severe symptoms	41 144 (90.3%)	39 303 (95.5%)	1 841 (4.5%)		1.00
Remitted	1 567 (3.4%)	1 342 (85.6%)	225 (14.4%)		3.58 (3.09, 4.15)
Incident	1 748 (3.8%)	1 323 (75.7%)	425 (24.3%)		6.86 (6.09, 7.74)
Persistent	1 083 (2.4%)	715 (66.0%)	368 (34.0%)		10.99 (9.61, 12.57)
Current self-rated emotional/mental health				< 0.0001	
Poor/ Fair/ Good	15 581 (34.2%)	13 505 (86.7%)	2 076 (13.3%)		5.73 (5.26, 6.24)
Very good/ Excellent	29 961 (65.8%)	29 178 (97.4%)	783 (2.6%)		1.00
Current self-rated emotional/mental health as compared to before March 2020				< 0.0001	
Worse	7 509 (16.5%)	6 483 (86.3%)	1 026 (13.7%)		3.62 (3.29, 3.94)
About the same	35 494 (77.9%)	34 007 (95.8%)	1 487 (4.2%)		1.00
Better	2 539 (5.6%)	2 193 (86.4%)	346 (13.6%)		3.61 (3.19, 4.09)
Drinking alcohol more often than March 2020					
Yes	7 487 (16.4%)	6 838 (91.3%)	649 (8.7%)		1.54 (1.41, 1.69)
No	38 055 (83.6%)	35 845 (94.2%)	2 210 (5.8%)		1.00
The presence of multimorbidity				< 0.0001	
None	13 055 (28.7%)	12 284 (94.1%)	771 (5.9%)		1.00
1–3 chronic physical conditions	29 780 (65.4%)	27 964 (93.9%)	1 816 (6.1%)		1.04 (0.95, 1.13)
≥ 4 chronic physical conditions	2 707 (5.9%)	2 435 (90.0%)	272 (10.0%)		1.78 (1.54, 2.06)

^§^ Pattern definition of moderate - severe symptoms (positive screen) of anxiety and depression at each survey: no positive screen for anxiety and depression at either survey; remitted (positive screen in 2018, but not 2020); incident (positive screen in 2020, but not in 2018); and persistent (positive screen in 2018 and 2020)

### Predisposing factors associated with MHSU

Associations between predisposing factors and MHSU controlling for enabling and need factors are presented in Table 2. In the overall sample, adults aged less than 44 years (aOR 3.02, 95% CI: 2.51, 3.62), 45 to 54 years (aOR 2.65 (2.34, 3.00) and 55 to 64 years (aOR: 1.84 (1.66, 2.05) were more likely to use mental health services than older adults aged ≥ 65 years. Respondents reporting past-year alcohol consumption less than (aOR: 0.75, 95% CI: 0.67, 0.85) and almost daily (aOR: 0.67, 95% CI: 0.56, 0.79) were also less likely to report MHSU than participants reporting no alcohol consumption in the past year. Females (aOR: 1.37, 95% CI: 1.24, 1.52) and other gender minority groups (aOR: 2.16, 95% CI: 1.16, 4.03) were more likely to report MHSU, as were participants reporting past-year cannabis use (aOR: 1.33, 95% CI: 1.20, 1.48).

**Table 2:** Multivariable analyses of mental health service use during COVID-19 by predisposing factors in the overall sample and by regional cohort

	Overall sample (n = 45 542)	British Columbia Generations Project (n = 13 786)	Alberta’s Tomorrow Project (n = 7 518)	Ontario Health Study (n = 9 652)	Quebec’s CARTaGENE (n = 5 186)	Atlantic PATH (n = 9 400)
	aOR (95% CI)*	aOR (95% CI)*	aOR (95% CI)*	aOR (95% CI)*	aOR (95% CI)*	aOR (95% CI)*
***Predisposing factors***						
CanPath Regional cohorts						
British Columbia Generations Project (BCGP)	**1.73 (1.41, 2.12)**					
Alberta’s Tomorrow Project (ATP)	1.07 (0.89, 1.28)					
Ontario Health Study (OHS)	**1.26 (1.02, 1.56)**					
Quebec’s CARTaGENE (CaG)	**1.00**					
Atlantic PATH	**1.39 (1.13, 1.72)**					
Age group						
< 44 years	**3.02 (2.51, 3.62)**	**4.15 (2.63, 6.56)**	**3.43 (2.27, 5.19)**	**2.73 (1.95, 3.81)**	-	**2.42 (1.68, 3.49)**
45–54 years	**2.65 (2.34, 3.00)**	**2.81 (2.26, 3.49)**	**3.02 (2.18, 4.19)**	**2.38 (1.80, 3.13)**	**2.47 (1.57, 3.87)**	**2.42 (1.85, 3.16)**
55–64 years	**1.84 (1.66, 2.05)**	**1.95 (1.64, 2.33)**	**1.89 (1.40, 2.55)**	**1.68 (1.32, 2.13)**	**1.90 (1.32, 2.73)**	**1.74 (1.37, 2.20)**
≥ 65 years	1.00	1.00	1.00	1.00	1.00	1.00
Gender identity						
Male	1.00	1.00	1.00	1.00	1.00	1.00
Female	**1.37 (1.24, 1.52)**	**1.23 (1.03, 1.47)**	1.25 (0.98, 1.58)	**2.00 (1.59, 2.52)**	1.08 (0.78, 1.48)	**1.35 (1.08, 1.70)**
Other gender minority	**2.16 (1.16, 4.03)**	1.34 (0.34, 5.36)	2.56 (0.48, 13.66)	2.03 (0.64, 6.49)	1.09 (0.14, 8.48)	**5.64 (1.52, 20.91)**
Race/ethnicity: Caucasian vs. non-Caucasian	1.12 (0.93, 1.35)	**1.50 (1.10, 2.04)**	1.06 (0.66, 1.71)	0.88 (0.64, 1.22)	0.76 (0.39, 1.47)	1.02 (0.51, 2.02)
Current smoking						
Yes vs. No	0.86 (0.71. 1.04)	1.05 (0.70, 1.58)	0.62 (0.37, 1.03)	0.72 (0.48, 1.09)	1.34 (0.83, 2.17)	0.81 (0.57, 1.15)
Past-year cannabis use						
Yes vs. No	**1.33 (1.20, 1.48)**	**1.23 (1.01, 1.49)**	**1.30 (1.01, 1.67)**	**1.47 (1.18, 1.83)**	1.12 (0.70, 1.80)	1.41 (1.15, 1.74)
Past-year average weekly alcohol consumption						
Never	1.00	1.00	1.00	1.00	1.00	1.00
Less than Daily (≤ 5 times/week)	**0.75 (0.67, 0.85)**	**0.74 (0.60, 0.92)**	0.79 (0.59, 1.05)	**0.64 (0.50, 0.83)**	**0.64 (0.40, 1.00)**	0.90 (0.70, 1.17)
Almost daily (Six to seven times a week	**0.67 (0.56, 0.79)**	**0.55 (0.41, 0.73)**	0.72 (0.46, 1.12)	0.72 (0.51, 1.03)	**0.50 (0.27, 0.93)**	0.84 (0.56, 1.26)
**Model C statistic**	**0.804**	**0.798**	**0.813**	**0.823**	**0.796**	**0.802**

*aOR, adjusted Odds Ratio for all other study variables including, predisposing, enabling and need factorsBolded estimates are statistically significant at p < 0.05

Stratified analyses by regional cohort showed similar results with respect to age, gender, and lifestyle behaviours. Regarding race/ethnicity, self-reporting as Caucasian was associated with increased MHSU (aOR: 1.50, 95% CI: 1.10, 2.04)- in the BCGP regional cohort.

### Enabling factors associated with MHSU

Associations between enabling factors and MHSU controlling for predisposing and need factors are presented in Table 3. In the overall sample, in comparison to respondents reporting an income of <$24,999 before the pandemic, participants reporting an income between $25,000 and $49,999 (aOR: 0.79, 95% CI: 0.63, 0.98), as well as preferring not to report their income (aOR: 0.71, 95% CI: 0.57, 0.88) were less likely to report MHSU. Respondents who reported having received informational/financial/practical support were more likely (aOR: 1.62, 95% CI: 1.48, 1.77) to report MHSU. Respondents who reported a delay in health care for a new health problem were not more likely to report MHSU than participants who did not report delays.

**Table 3:** Multivariable analyses of mental health service use during COVID-19 by enabling factors in the overall sample and by regional cohort

	Overall sample (n = 45 542)	British Columbia Generations Project (n = 13 786)	Alberta’s Tomorrow Project (n = 7 518)	Ontario Health Study (n = 9 652)	Quebec’s CARTaGENE (n = 5 186)	Atlantic PATH (n = 9 400)
	aOR (95% CI)*	aOR (95% CI)*	aOR (95% CI)*	aOR (95% CI)*	aOR (95% CI)*	aOR (95% CI)*
***Enabling factors***						
Total household income in year prior to pandemic						
< $24,999	1.00	1.00	1.00	1.00	1.00	1.00
$25,000 - $49,999	**0.79 (0.63, 0.98)**	0.95 (0.64, 1.41)	0.68 (0.38, 1.23)	0.91 (0.56, 1.46)	**0.51 (0.27, 0.98)**	0.70 (0.43, 1.13)
$50,000 - $74,999	0.83 (0.67, 1.03)	0.88 (0.60, 1.30)	0.88 (0.51, 1.51)	0.73 (0.46, 1.17)	0.57 (0.31, 1.06)	0.95 (0.61, 150)
$75,000 - $99,999	0.83 (0.66, 1.02)	0.97 (0.66, 1.43)	0.69 (0.40, 1.19)	0.92 (0.58, 1.46)	**0.48 (0.25, 0.93)**	0.80 (0.50, 1.28)
$100,000 - $149,999	0.88 (0.71, 1.08)	1.04 (0.71, 1.53)	0.77 (0.45, 1.31)	0.86 (0.55, 1.36)	0.64 (0.34, 1.21)	0.87 (0.55, 1.38)
≥ $150,000	0.87 (0.70, 1.08)	1.14 (0.77, 1.69)	0.75 (0.44, 1.27)	0.87 (0.55, 1.38)	0.53 (0.26, 1.05)	0.80 (0.50, 1.29)
Prefer not to answer	**0.71 (0.57, 0.88)**	0.82 (0.55, 1.21)	**0.46 (0.26, 0.82)**	0.68 (0.42, 1.11)	0.76 (0.38, 1.50)	0.82 (0.51, 1.32)
Missing response	0.90 (0.59, 1.37)	0.62 (0.27, 1.41)	1.49 (0.55, 4.07)	0.51 (0.14, 1.86)	1.10 (0.44, 2.78)	1.04 (0.81, 1.24)
Decrease in income during pandemic						
Yes vs. No	0.92 (0.84, 1.01)	0.99 (0.84, 1.17)	0.87 (0.70, 1.08)	**0.81 (0.66, 1.00)**	0.83 (0.59, 1.18)	1.00 (0.81, 1.24)
Medical or other professional with exposure to patients						
Yes vs. No	1.03 (0.89, 1.20)	0.92 (0.69, 1.22)	1.13 (0.83, 1.53)	1.22 (0.84, 1.77)	0.90 (0.49, 1.63)	0.98 (0.71, 1.34)
Essential worker with exposure to public						
Yes vs. No	0.92 (0.79, 1.07)	0.93 (0.72, 1.20)	1.16 (0.82, 1.53)	1.12 (0.74, 1.68)	0.60 (0.32, 1.11)	0.73 (0.52, 1.02)
Informational/financial/practical support received						
Yes vs. No	**1.62 (1.48, 1.77)**	**1.53 (1.31, 1.80)**	**1.66 (1.34, 5.06)**	**1.85 (1.53, 2.23)**	**1.62 (1.19, 2.22)**	**1.46 (1.20, 1.77)**
Delay in health care for new health problem						
Yes	0.90 (0.75, 1.10)	0.58 (0.29, 1.15)	1.04 (0.79, 1.70)	0.51 (0.22, 1.21)	1.38 (0.79, 2.41)	0.69 (0.28, 1.73)
No	1.00	1.00	1.00	1.00	1.00	1.00
Not applicable/not answered	**0.75 (0.62, 0.91)**	**0.50 (0.25, 0.97)**	-	0.49 (0.21, 1.13)	**0.67 (0.48, 0.94)**	0.54 (0.22, 1.33)
**Model C statistic**	**0.804**	**0.798**	**0.813**	**0.823**	**0.796**	**0.802**

*aOR, adjusted Odds Ratio for all other study variables, including, predisposing, enabling and need factorsBolded estimates are statistically significant at p < 0.05

Stratified analyses by regional cohort showed similar results in associations between MHSU and receipt of informational/financial/practical support, as well as delays in health care for a new health problem. Findings did not show an association between income and MSHU in the BCGP, OHS, and Atlantic PATH regional cohorts, whereas differences were observed in the CaG and ATP regional cohorts. In Ontario, respondents who reported a decrease in income during the pandemic (aOR: 0.81, 95% CI: 0.66, 1.00) were less likely to report MHSU.

### Need factors associated with MHSU

Associations between need factors and MHSU after controlling for predisposing and enabling factors are presented in Table 4. With respect to patterns of moderate to severe symptoms of anxiety, respondents with remitted (aOR: 1.30, 95% CI: 1.10, 1.55), incident (aOR: 1.81, 95% CI: 1.55, 2.10) and persistent (aOR: 1.89, 95% CI: 1.54, 2.31) symptoms were more likely to consult than participants with no symptoms of anxiety reaching that threshold. Similarly, respondents with remitted (aOR: 1.66, 95% CI: 1.39, 1.98), incident (aOR: 2.03, 95% CI: 1.75, 2.36) and persistent (aOR: 2.83, 95% CI: 2.36, 3.78) moderate to severe symptoms of depression were more likely to consult than participants with no symptoms of depression reaching that threshold. Respondents who self-rated their mental health as poor/fair/good were more likely to consult than participants reporting their mental health as very good or excellent (aOR 3.18, 95% CI: 2.88, 3.51). Respondents who reported a better mental health in comparison to a similar mental health prior to the pandemic were more likely (aOR: 2.40, 95% CI: 2.09, 2.75) to report MHSU. Compared to respondents reporting no chronic physical conditions, individuals reporting four or more chronic related physical conditions (aOR: 1.27, 95% CI: 1.07, 1.50) were more likely to report MHSU.

**Table 4:** Multivariable analyses of mental health service use during COVID-19 by need factors in the overall sample and by regional cohort

	Overall sample (n = 45 542)	British Columbia Generations Project (n = 13 786)	Alberta’s Tomorrow Project (n = 7 518)	Ontario Health Study (n = 9 652)	Quebec’s CARTaGENE (n = 5 186)	Atlantic PATH (n = 9 400)
	aOR (95% CI)*	aOR (95% CI)*	aOR (95% CI)*	aOR (95% CI)*	aOR (95% CI)*	aOR (95% CI)*
***Need factors***						
Pattern of moderate – severe symptoms of anxiety^§^						
No moderate-severe symptoms	1.00	1.00	1.00	1.00	1.00	1.00
Remitted	**1.30 (1.10, 1.55)**	**1.43 (1.05, 1.95)**	**1.48 (1.03, 2.15)**	1.12 (0.76, 1.65)	1.56 (0.80, 3.05)	1.04 (0.70, 1.53)
Incident	**1.81 (1.55, 2.10)**	**2.07 (1.58, 2.70)**	**1.73 (1.20, 2.50)**	**1.91 (1.39, 2.62)**	1.48 (0.87, 2.51)	**1.59 (1.14, 2.22)**
Persistent	**1.89 (1.54, 2.31)**	**2.22 (1.52, 3.26)**	**1.92 (1.20, 3.05)**	1.30 (0.84, 1.99)	1.65 (0.72, 3.78)	**2.22 (1.45, 3.40)**
Pattern of moderate – severe symptoms of depression^§^						
No moderate-severe symptoms	1.00	1.00	1.00	1.00	1.00	1.00
Remitted	**1.66 (1.39, 1.98)**	**1.41 (1.02, 1.94)**	**1.61 (1.06, 2.44)**	**1.95 (1.35, 2.82)**	**1.51 (0.80, 2.84)**	**2.03 (1.39, 2.96)**
Incident	**2.03 (1.75, 2.36)**	**2.53 (1.94, 3.30)**	**1.61 (1.12, 2.31)**	**1.75 (1.27, 2.42)**	**2.49 (1.48, 4.19)**	**2.00 (1.44, 2.78)**
Persistent	**2.83 (2.36, 3.78)**	**2.90 (2.07, 4.07)**	**2.62 (1.78, 3.87)**	**3.04 (2.09, 4.43)**	**2.43 (1.09, 5.43)**	**2.79 (1.89, 4.12)**
Current self-rated emotional/mental health						
Poor/ Fair/ Good vs. Very good/ Excellent	**3.18 (2.88, 3.51)**	**2.92 (2.46, 3.46)**	**3.40 (2.67, 4.35)**	**3.48 (2.77, 4.36)**	**2.55 (1.83, 3.55)**	**3.41 (2.76, 4.21)**
Current self-rated emotional/mental health as compared to before March 2020						
Worse	1.09 (0.99, 1.21)	1.00 (0.84, 1.20)	1.16 (0.91, 1.49)	1.03 (0.83, 1.29)	1.42 (0.97, 2.08)	1.15 (0.92, 1.44)
About the same	1.00	1.00	1.00	1.00	1.00	1.00
Better	**2.40 (2.09, 2.75)**	**2.35 (1.86, 2.98)**	**2.59 (1.82, 3.67)**	**1.85 (1.34, 2.56)**	**3.08 (1.89, 5.02)**	**2.56 (1.95, 3.37)**
Drinking alcohol more often than March 2020						
Yes vs. No	1.05 (0.95, 1.16)	1.06 (0.89, 1.28)	1.04 (0.80, 1.34)	1.17 (0.94, 1.47)	1.25 (0.88, 1.77)	0.86 (0.68, 1.10)
The presence of multimorbidity						
None	1.00	1.00	1.00	1.00	1.00	1.00
1–3 chronic physical conditions	1.05 (0.96, 1.16)	1.08 (0.91, 1.28)	1.09 (0.87, 1.36)	1.13 (0.91, 1.41)	0.99 (0.72, 1.37)	0.94 (0.76, 1.15)
≥ 4 chronic physical conditions	**1.27 (1.07, 1.50)**	**1.42 (1.06, 1.89)**	1.42 (0.91, 2.23)	1.08 (0.75, 1.55)	1.74 (0.90, 3.37)	1.18 (0.83, 1.67)
**Model C statistic**	**0.804**	**0.798**	**0.813**	**0.823**	**0.796**	**0.802**

*aOR, adjusted Odds Ratio for all other study variables, including, predisposing, enabling and need factorsBolded estimates are statistically significant at p < 0.05^§^ Pattern definition of moderate - severe symptoms (positive screen) of anxiety and depression at each survey: no positive screen for anxiety and depression at either survey; remitted (positive screen in 2018, but not 2020); incident (positive screen in 2020, but not in 2018); and persistent (positive screen in 2018 and 2020)

Stratified analyses by regional cohort showed similar results in MHSU in relation to patterns of moderate to severe symptoms of depression and anxiety, as well as current emotional and mental health and change in mental health from before the pandemic.

## Discussion

This study captured information early in the COVID-19 pandemic (May to December 2020) and found that 6.3% of respondents reported MHSU. This estimate is lower than the 13.5% reported in a Canadian survey [29] carried out between October and December 2020. The higher estimate in the latter study may in part be explained by the fact that it included twice and five times the percentage of individuals with moderate/severe depression and anxiety, respectively.

Study findings showed the importance of need factors in explaining MHSU, controlling for predisposing and enabling factors, which has been previously reported [16]. Perceived mental health status and presence of anxiety and depression symptoms have been associated with increased MHSU [1, 16]. To our knowledge, there are no general population studies that have looked at MHSU and changes in self-reported mental health status. We observed that individuals reporting no change in their mental health, during as compared to prior the pandemic, were not more likely to report MHSU, whereas individuals with an improvement in their mental health were more likely to report MHSU. A Canadian survey during the second wave of the pandemic showed a higher proportion of individuals aged 35 to 64 years reporting poor or fair mental health as compared to the year prior to the pandemic during which, up to 25% reported a mental health need for care [30]. Although the cross-sectional nature of the current study design precludes concluding on causality, these findings suggest the importance of receipt of mental health services in improving perceived mental health. In a recent longitudinal study in community primary care older adults, minimally adequate care for depression and anxiety was associated with improved self-reported mental health outcomes, such as life satisfaction and health related quality of life [31].

In this study, close to one in five respondents reported an increase in alcohol consumption from prior to the pandemic, and this was not associated with MHSU. In general, individuals with alcohol-related problems are not more likely to report MHSU [32], but when they do use health care services, it is usually for the physical problems related to alcohol consumption [33]. Canadian surveys conducted between April and December 2020 showed that between 28% and 32% of respondents reported an increase in alcohol consumption [3, 29]. When reporting on older adults specifically, only 20% had reported an increase in either alcohol or cannabis use [29]. The lower rate observed in this study sample may be explained that it was comprised of a larger proportion of older adults. The observations on need factors were similar for each regional cohort studied. As evidenced by a systematic review on the predisposing, enabling, and need factors, the latter were the most consistently associated with MHSU [16].

Overall, among the enabling factors studied, controlling for need and predisposing factors, receipt of informational/financial/practical support was associated with increased MHSU, similarly reported in a previous Canadian population-based study [32]. When looking at reported income in the year prior to the pandemic, in general, findings from this study did not show significant income-related inequalities in MHSU [34]. In the few instances where income was associated with MHSU, differences observed did not favor participants who reported higher income levels. In previous Canadian population-based studies, income was not associated with overall MHSU and specifically with use of general practitioners and mental health specialists, such as psychiatrists [1, 32]. Canada has a public health system where residents are covered for the majority of their medical consultations with physicians. Consultations with health professionals working in the private sector would therefore not be covered under the public health system. A decrease in income during the pandemic was marginally associated with a reduced likelihood of MHSU in the overall sample and significantly in the Ontario Health Study. This finding may suggest the presence of socio-economic barriers in mental health care access related to job loss and employee health benefit coverage. In fact, during the second wave of the pandemic, about one in 5 Canadians reported a mental health need, with counselling or therapy being the most common [30]. Barriers to accessing these services included, among others, income and lack of insurance coverage [30]. Population studies prior to the pandemic also showed that income was associated with consulting mental health professionals [32] and that out-of-pocket costs related to psychological services were a barrier in receiving psychotherapy [35]. Income-based inequities in the use of psychological services and psychotherapy reported in Canada are also greater than those observed in other countries with a general practitioner gatekeeper system [23]. Future research should focus on better describing the reasons for the absence of mental health service use. This may help better identify the ‘health care system and individual circumstances’ that act as barriers to mental health care [36].

Among predisposing factors studied and after controlling for enabling and need factors, age and gender, as well as alcohol consumption and cannabis use were associated with MHSU. Older adults aged 65 years and over were less likely to consult than other age groups, a finding similarly reported during the pandemic in Canada [10] and elsewhere [11, 37]. Increased resilience mitigating the mental health effects of COVID-19 in older adults has been put forth as a possible explanation [38]. Another Canadian survey conducted during the pandemic also showed better coping skills and mental health in older adults in comparison to younger adults [29]. Alternatively, the decreased MHSU observed may in part be related to fear of infection, also evidenced by many older adults interrupting their home care services due to this reason [39]. Similarly to what has been reported elsewhere [10], females and other gender minority groups were more likely to use mental health services even after controlling for need factors.

Further, lifestyle behaviours such as alcohol consumption in the current study was associated with reduced MHSU, which has been previously reported in other Canadian studies [2, 32]. These findings may also reflect the fact that daily alcohol consumption was found to be more prevalent in adults aged 45 years and over (14.4%) as compared to younger adults aged 35 to 44 years (5.8%), and these adults aged 45 years and over were also less likely to report MHSU (5.9% vs. 15.9%). Additional post-hoc stratified analyses by age group showed that among the adults aged 35–44 years, respondents reporting less than daily and almost daily alcohol consumption were not more likely to report MHSU, as compared to those not reporting past-year alcohol consumption. Among participants aged 45 years and over, respondents reporting less than daily and almost daily alcohol consumption were less likely to report MHSU, as compared to respondents not reporting past-year alcohol consumption. Studies have similarly shown that adults aged 55 years and over are less likely to report problematic substance use disorders [29, 40] and consequently less likely to consult mental health services. The current observation where alcohol consumption is associated with lower MHSU may also suggest the presence of maladaptive behaviours during the pandemic and the presence of unmet mental health care need. In fact, when looking at the need factor, participants reporting an increase in alcohol consumption since March 2020 were not more likely to consult for mental health reasons. In this study, however, past-year cannabis use was associated with increased MHSU, with a larger proportion from the younger age group. Other studies show that two out of five users of cannabis also reported increased and problematic use during the pandemic [29].

## Limitations

Data used in this study were from a sample of adults and older adults participating in one of CanPath’s longitudinal regional cohorts. Consequently, these individuals may not be representative of the general Canadian population in relation to social and health status and access to health care services. CanPath participants were more likely to self-report as White, female, retired, and report higher income and education than the general Canadian population [25]. Further, we could not distinguish between MHSU in the public versus private sector and respective associated factors.

## Conclusion

This pan-Canadian study highlights that need and predisposing factors were the most consistent in explaining MHSU during the pandemic context. In general, socio-economic inequities, including income reported during the pandemic in relation to MHSU were not observed after controlling for predisposing and need factors. However, the observation that a reduction in income may be associated with a reduced likelihood of MHSU underlines the importance of health policies aimed at improving access and insurance coverage to mental health care professionals in primary care. Pandemic effects leading to changes in income that may potentially influence longer term socio-economic barriers in accessing mental health services warrants more research. To this, contextual factors including health policies increasing access to mental health services and psychological therapies for residents as was the case in Ontario and Manitoba during the pandemic [41] would be important to consider in future studies. Finally, other contextual enabling factors (e.g. mental health care expenditures, health service facilities and human resources available) [42] at the community level need further consideration.

## Data Availability

The authors are not legally authorised to share or publicly publish CanPath data. Participants were not requested to give informed consent for data sharing. Requests for access to the data should be addressed to CanPath.
